# High Efficacy of Triclabendazole/Ivermectin Combination Compared to Triclabendazole Monotherapy for Treating Human Fascioliasis in Upper Egypt: A Prospective Study

**DOI:** 10.3390/tropicalmed10080221

**Published:** 2025-08-06

**Authors:** Waleed Attia Hassan, Haidi Karam-Allah Ramadan, Mona Gaber, Samia S. Alkhalil, Alzahraa Abdelraouf Ahmad

**Affiliations:** 1 Department of Tropical Medicine and Gastroenterology, Faculty of Medicine, Assiut University, Assiut 71515, Egypt; wallo403a@aun.edu.eg; 2 Department of Medical Parasitology, Faculty of Medicine, Assiut University, Assiut 71515, Egypt; m.g.mostafa@aun.edu.eg (M.G.); zahraaabdelraouf@aun.edu.eg (A.A.A.); 3 Department of Medical Laboratory Sciences, College of Applied Medical Sciences, Shaqra University, Alquwayiyah, Riyadh 11961, Saudi Arabia; salkhalil@su.edu.sa

**Keywords:** human fascioliasis, treatment failure, triclabendazole, ivermectin, combination therapy, adjunctive treatment

## Abstract

Triclabendazole (TCBZ) is the gold standard treatment for fascioliasis. However, reports on resistance are increasing, emphasizing the need for alternative therapy. Combining TCBZ with ivermectin (IVM) was found to be effective for treating animal fascioliasis. Building on this, we aimed to evaluate the efficacy of the TCBZ/IVM combination therapy for human fascioliasis. This study enrolled 136 patients with *Fasciola* from Upper Egypt, and they were divided into the first group (*n* = 65), who received TCBZ monotherapy, and the second group (*n* = 71), who received the TCBZ/IVM combination. Assessments were to evaluate treatment response based on clinical, eosinophilic, and radiological parameters. Chronic fasciolosis was diagnosed in 17 patients (12.5%). No differences were observed in age and sex. Significant improvements were noted in all parameters in both groups, with more pronounced effects observed in the second group. A significantly higher complete response, including clinical, eosinophilic, and radiological improvements, was reported in the combined therapy group, with 53.3% compared to 26.2% in the monotherapy group (*p* < 0.001). A high baseline eosinophilic count was significantly associated with response. The efficacy of the TCBZ/IVM combination for treating human fascioliasis suggested a possible boosting effect, which can benefit regions of TCBZ failure. Further large-scale randomized studies are warranted to confirm these findings.

## 1. Introduction

Fascioliasis is a significant zoonotic disease caused by liver flukes of the genus *Fasciola*, specifically *Fasciola hepatica*, *Fasciola gigantica*, or both of them as a hybrid infection. The disease affects both humans and livestock worldwide, with approximately 17 million individuals affected across 81 different countries [1]. Fascioliasis has been prioritized by the World Health Organization (WHO) in its roadmap for addressing Neglected Tropical Diseases (NTDs) by 2030 [2]. The disease is presented in two clinical phases: acute and chronic phases, whereas the chronic phase is associated with biliary obstruction and fibrosis [3].

Fascioliasis is prevalent in areas with extensive agricultural and livestock farming practices, where people are often exposed to raw vegetables containing the encysted metacercaria. Notably, the prevalence of this condition has been reported in Bolivia, Peru, Cuba, China, Iran, Vietnam, and Egypt [4]. In Egypt, both *F. hepatica* and *F. gigantica* coexist, and *F. hepatica* is the more prevalent species found in humans, while both species have been retrieved from various animal species [5,6]. In the past few decades, fascioliasis has been recognized as an endemic disease with a re-emerging condition in Upper Egypt [7,8]. Human cases range from mild to severe, particularly in the Nile Delta region [9]. Meanwhile, Upper Egypt experiences fewer cases, but they are rising because of climate change [10].

Triclabendazole, a derivative of benzimidazole, has been recognized as the standard treatment for fascioliasis because of its effectiveness in targeting both immature and adult flukes [11]. Several reports of TCBZ resistance and treatment failures in humans have also been published, raising concerns about the long-term utility of TCBZ [7,12,13,14,15]. These challenges have driven the search for alternative or adjunctive treatments to improve treatment effectiveness and overcome resistance.

Ivermectin (IVM) is an effective broad-spectrum antiparasitic agent, exhibiting significant efficacy against various helminths and ectoparasites through a distinct mechanism of action that differs from TCBZ, which targets the nervous and muscular systems of helminths [16,17,18]. IVM has demonstrated safety and efficacy in treating various nematode infections, including onchocerciasis, strongyloidiasis, and enterobiasis, at doses of 150–200 μg/kg, and lymphatic filariasis at 400 μg/kg [19]. Although IVM is not fully effective against trematodes, it was tested in combination therapies as a trial to broaden the activity spectrum of other anthelmintics [20].

In veterinary medicine, the use of a combination of drugs is a common approach to managing parasitic infections in livestock, providing several benefits over single-drug treatments, such as targeting mixed infections involving nematodes, flukes, and even ectoparasites [21]. TCBZ is commonly marketed along with other anthelmintics, including levamisole (LEV), oxfendazole (OXF), ivermectin (IVM), abamectin, and moxidectin (MOX). These combinations can have additive or synergistic effects, allowing for lower drug concentrations, thus reducing environmental impact and selection pressure for resistance [21,22]. Furthermore, drug combinations can improve pharmacokinetics and pharmacodynamics, extend the effective lifespan of the drugs involved, enhance drug bioavailability, and potentially delay the onset of drug resistance [22,23,24].

Based on preliminary findings from in vitro and in vivo animal studies, IVM demonstrates potential as a promising candidate in a combination therapy approach for controlling fascioliasis. Notably, a synergistic effect exceeding a 27% increase in ovicidal activity was observed against *F. hepatica* eggs when IVM was combined with other treatments [25]. Furthermore, combination therapies incorporating IVM, such as those with TCBZ [26] or closantel [27], have exhibited effectiveness in managing animal fascioliasis [25]. Combining IVM and TCBZ showed a synergistic value through utilizing distinct mechanisms of action, where TCBZ impairs nutrient absorption and damages the parasite’s tegument, while IVM induces worm paralysis by targeting chloride channels in nerves and muscles [28,29]. This synergy may improve drug efficacy and reduce resistance risk by targeting parasites through multiple pathways simultaneously [30]. Additionally, a previous study demonstrated that IVM modulates P-glycoprotein activity, reducing TCBZ efflux from resistant flukes. Consequently, higher TCBZ concentrations were recovered from the resistant liver flukes when IVM was present [31].

This raises the question of the potential efficacy of this drug combination for treating human fascioliasis, which has not been previously explored. Establishing the safety profile, dosing guidelines for IVM in human parasitic diseases, such as strongyloidiasis, lymphatic filariasis, and scabies [32], and pharmacological efficacy observed in animal studies have suggested its potential use in treating fascioliasis, especially in areas where resistance to TCBZ or treatment failure is challenging. Therefore, this study aimed to address a gap in the literature by evaluating the effectiveness of TCBZ/IVM combination therapy for treating human fascioliasis.

## 2. Methods

### 2.1. Patient Recruitment and Diagnosis

This study was an open-label, nonrandomized prospective study conducted at the outpatient clinic of Al-Rajhy Liver University Hospital in Assiut, Upper Egypt, between June 2023 and August 2024. This hospital is situated in Assiut Governorate, in Upper Egypt, and serves numerous nearby governorates as a major tertiary hospital. Patients included in this study met the case definition for fascioliasis infection [33] and were residents of the *Fasciola* endemic region in Assiut Governorate, in Upper Egypt, which is located on the Nile’s west bank. It is mainly a rural community where farming is the primary occupation, with high reliance on agriculture and livestock. Participants with fascioliasis were enrolled in the study, but those with evidence of other parasitic infections or who had previously received other anti-parasitic medications, or subjects with known hypersensitivity to any of the tested medications, were excluded.

The diagnosis of fascioliasis was based on three key parameters: clinical evaluation and laboratory and/or radiological findings. The clinical manifestations included fever, right hypochondrial or epigastric pain, nausea, vomiting, jaundice, dark-colored urine, or itching. Laboratory assessment included detection of positive anti-*Fasciola* antibodies, elevated eosinophil levels (>6%), and/or the detection of *Fasciola* eggs on stool microscopy. Radiological diagnosis included the detection of hepatic focal lesions (HFLs) on abdominal ultrasound or computed tomography scans. These lesions result from the migration of juvenile flukes through liver tissue, causing localized inflammation, necrosis, and fibrosis. Bile duct abnormalities, such as duct dilatation and crescent-shaped structures, may also be evident [34].

Laboratory tests were conducted on all recruited patients, including complete blood count (CBC), including eosinophil percentage and absolute eosinophil count; liver enzymes; and stool examinations (involving direct smear and formal-ether concentration methods) to detect *Fasciola* eggs and exclude other parasitic infections [35]. A serological analysis was conducted using the Indirect Hemagglutination Assay (IHA) with a Distomatose Fumouze IHT kit (Fumouze Diagnostic Laboratories, Levallois-Perret, France). Anti-*Fasciola* antibody titers were measured, with a titer of 1:320 considered positive for comparison.

### 2.2. Treatment Regimens and Follow-Up

The recruited patients were categorized into two cohorts; the first group received TCBZ monotherapy, while the second group received a combination therapy of TCBZ and IVM. TCBZ was administered according to the WHO protocol as a double dose of 10 mg/kg per dose, 12 h apart with a fatty meal (Egaten, Novartis Pharma AG, Basel, Switzerland) [11]. IVM was administered at a single dose of 200 µg/kg, consistent with the dosages used for treating human nematode infections, such as strongyloidiasis, lymphatic filariasis, and scabies [18]. Newly diagnosed patients were enrolled consecutively throughout the study period. Treatments were distributed randomly among the patients. Both TCBZ and IVM were administered under direct observation to ensure adherence to the treatment protocol. Follow-up was performed after two months of receiving therapy to evaluate the cure response based on clinical improvement, eosinophil response, and radiological findings. According to the WHO, the persistence of clinical manifestations, along with either high eosinophilia (>6% in peripheral blood) or HFLs, is considered an indicator of treatment failure with TCBZ [11].

### 2.3. Statistical Analysis

It was performed using SPSS (version 21). For categorical data, frequency distributions and percentages were calculated, whereas quantitative variables were presented using mean ± standard deviation or median (interquartile range; IQR), contingent upon the data distribution. Comparisons between categorical variables were conducted using the chi-square or Fisher’s exact test based on sample size and expected frequencies. Quantitative variables were compared between the two groups using the independent samples t-test and Mann–Whitney U test. The Wilcoxon signed-rank and McNemar test were used to analyze within-group comparisons of data before and after treatment. To identify factors associated with therapeutic response, multivariate regression analysis was performed. A *p*-value of less than 0.05 was considered statistically significant.

## 3. Results

### 3.1. Characteristics of the Study Population

A total of 136 patients were enrolled in the study; 65 patients received TCBZ monotherapy, and 71 patients received a combination of TCBZ and IVM. As shown in Table 1, the baseline characteristics of the participants demonstrated no significant differences between the two groups in terms of gender, age, or residence. The study population was predominantly female (*n* = 77, 56.6%) and from rural areas (*n* = 100, 73.5%). The mean anti-*Fasciola* antibody titer was notably higher in the TCBZ monotherapy group (960 ± 396). The median duration of symptoms was 17 (20) days. Chronic fascioliasis was diagnosed in 17 cases (12.5%) based on the identification of *Fasciola* ova in stool samples and the detection of adult worms, causing biliary obstruction in the biliary channels through imaging techniques.

### 3.2. Clinical, Laboratory, and Radiological Data of the Participants

**Figure 1:**
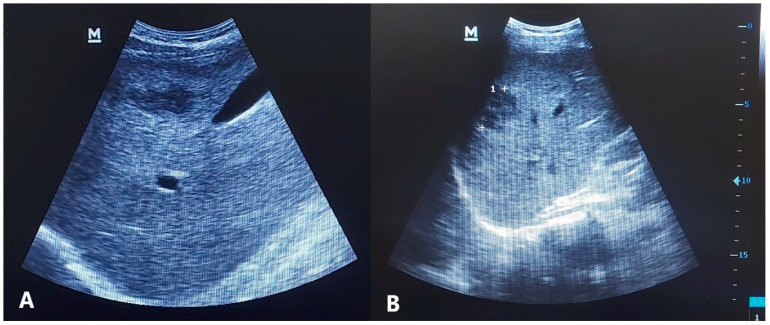
Pattern of different hypoechoic hepatic focal lesions by ultrasound in two patients (**A**,**B**) at enrollment in the study.

**Figure 2:**
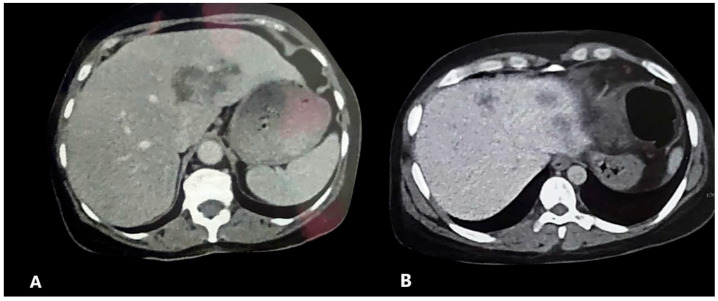
Pattern of different hypointense hepatic focal lesions by contrast-enhanced computed tomography in two patients (**A**,**B**) at enrollment in the study.

Significant improvements were observed in all evaluated parameters in both treatment groups, except for the presence of *Fasciola* ova in stool samples, which showed no notable difference between the groups. Clinically, fever and abdominal pain were reduced following treatment. Regarding laboratory findings, the liver enzymes, median white blood cell (WBC) count, eosinophil count, and percentages showed significant improvement. Radiological assessments showed that HFLs were present in 25 patients in the monotherapy group and in 41 patients in the combination therapy group (Figure 1 and Figure 2). After the treatment, using the same modality and clinician to reduce test variability, a significant reduction in the percentages of HFLs was observed in both groups. In the monotherapy group, the number of HFLs decreased from 25 to 8, whereas in the combination therapy group, it decreased from 41 to 6 (Table 2).

### 3.3. Evaluation of Treatment Response

As depicted in Table 3, the study findings indicate that after two months of patient follow-up, there was a significant improvement in all evaluated parameters—clinical, eosinophilic, and radiological—in both cohort groups. The combined therapy group exhibited a more pronounced positive effect with statistically significant differences. A complete response, defined as simultaneous improvement in all three parameters within the same patient, was reported in 40 out of 71 patients (53.3%) in the combined therapy group compared with only 17 patients (26.2%) in the monotherapy group (*p* < 0.001). In the combined therapy group, two cases developed mild itching, and ten cases had transient mild nausea and epigastric pain.

### 3.4. Factors Associated with Treatment Response

The analysis of factors associated with a complete response in patients treated with TCBZ monotherapy and combined TCBZ + IVM therapy showed that the most notable finding was the significant association between a high eosinophil count at baseline and a complete response (*p* = 0.009; OR = 1). This suggests that patients with higher eosinophil counts at the start of treatment are more likely to achieve a complete response. However, the type of treatment (TCBZ monotherapy vs. TCBZ + IVM) did not significantly affect the complete response (*p* = 0.168; OR = 1.860) (Table 4). Other variables such as age, gender, residence, duration of symptoms, and presence of HFL at baseline did not significantly influence the complete response.

## 4. Discussion

TCBZ has been the standard therapy and control of fascioliasis in several countries, including Egypt, since 1998 [11]. It exerts its action on both early immature and adult *Fasciola* flukes and disrupts microtubule formation and energy metabolism [36]. Despite its effectiveness, there are increasing reports of growing resistance to TCBZ in *Fasciola* populations, which has resulted in treatment failures in both livestock and humans [7,15,37].

In this study, the recruited patients were mostly female and predominantly from rural areas. This reflects demographic patterns commonly observed in fascioliasis-endemic regions, consistent with previous studies in Egypt [9,38,39,40]. The median duration of symptoms was 17 days, and the mean *Fasciola* antibody titer at baseline was consistent with the acute to subacute presentation of the disease [41]. Most reported cases were in the acute phase of the disease, whereas chronic fascioliasis was diagnosed in only 12.5% of cases, which is consistent with previous studies conducted in the same region [42].

The results of this study provide valuable insights into the effectiveness of combining TCBZ and IVM for treating human fascioliasis in Upper Egypt. The findings indicate a significant improvement in clinical, laboratory, and radiological findings in the combination therapy group. Patients who received combined therapy showed a significant increase in each of these parameters, along with higher rates of complete responses. These encouraging results suggest that combination therapy could play a crucial role in addressing TCBZ resistance and enhancing overall treatment outcomes.

The significant improvements observed in fever, abdominal pain, and ascites, along with the reduction in HFLs, underscore the effectiveness of both treatment regimens in managing the disease. Additionally, the decreases in liver enzyme levels, WBC counts, eosinophil counts, and percentages support this effectiveness. These findings align with previously reported results highlighting the efficacy of TCBZ in reducing the laboratory parameters and imaging profile of fascioliasis infection [43].

TCBZ effectively eliminates the parasite, which in turn reduces inflammation, tissue damage, and the mechanical harm caused by the migration and presence of flukes in the liver and biliary system. TCBZ functions by inhibiting the polymerization of tubulin in the parasite’s cytoskeleton, disrupting cellular division and energy metabolism, ultimately leading to the parasite’s death [36,44]. This mechanism of action allows TCBZ to target both the immature and adult stages of the parasite, making it particularly effective for treating both acute and chronic fascioliasis [11].

Our findings demonstrate that TCBZ monotherapy achieved a complete cure in only 26.2% (17/65) of patients after two months, whereas 73.8% (48/65) showed no therapeutic response. In comparison, the combination therapy group exhibited a significantly higher cure rate of 53.3%, suggesting potential treatment inefficacy of TCBZ alone under these conditions. This is in concordance with a recent study, which compared the TCBZ/IVM combination with other drugs and reported a percentage of efficacy regarding egg reduction to be 98.8% [45].

In line with our results, Hien et al. reported similarly low cure rates of 36% for TCBZ monotherapy, assessed through various outcome measures, including clinical, laboratory, and imaging parameters [33]. Additionally, Terashima et al. noted that fascioliasis failed to respond to standard-of-care (SOC) doses of TCBZ in 21 out of 27 patients who had not responded to at least two doses of TCBZ at a dosage of 10 mg/kg [46]. This situation underscores the known challenges in the management of fascioliasis, particularly regarding persistent inflammation (eosinophilia) and tissue damage associated with the disease [43]. In contrast, previous studies conducted in Egypt and other countries have reported higher response rates to TCBZ, ranging from 69% to 96%. These discrepancies may be attributable to variations in diagnostic criteria or disease stage, whether acute or chronic fascioliasis, or differences in biotype/genetics of the flukes [47,48,49].

TCBZ is commonly co-administered with other anthelmintics in veterinary practice to produce adjunctive or synergistic effects, allowing for reduced dosages, improving drug bioavailability, and prolonging treatment efficacy [21,22,23]. IVM is a broad-spectrum macrolactone that is not commonly used to treat fascioliasis because it is primarily effective against nematodes and ectoparasites. However, IVM was previously used in combination with other medications, such as TCBZ, albendazole, nitroxynil, clorsulon, and closantel, for the treatment of liver fluke infections in cattle and demonstrated remarkable effects [50].

A previous report demonstrated that combined IVM and TCBZ therapy in cattle and sheep significantly reduces *Fasciola* infestations [26]. A previous clinical trial in sheep found that co-administration of these compounds improves their pharmacokinetic properties, and higher peak plasma concentrations of TCBZ metabolites were detected after the co-administration of TCBZ and IVM compared to those obtained following TCBZ treatment in isolation [51]. Moreover, another study reported that the ivermectin-induced modulation of P-glycoprotein activity decreased TCBZ efflux from the resistant liver flukes, and higher concentrations of TCBZ were recovered from the resistant liver flukes in the presence of ivermectin [31].

IVM modulates the immune response, owing to its anti-inflammatory properties, in addition to its ability to promote healing [52]. It can reduce the production of pro-inflammatory cytokines, particularly Th2 cytokines such as interleukin-6 (IL-6) and tumor necrosis factor-alpha (TNF-α), as well as interleukin-5 (IL-5), which is a key factor in the production of eosinophils [53]. This reduction may help decrease tissue damage, which could explain the significant improvements observed in clinical outcomes, eosinophil levels, and radiological indicators within the combined therapy group in the current study.

There are only a few reports of the direct effects of IVM on adult *Fasciola* species [54]. Recent research by Romero-Neto and colleagues demonstrated the ovicidal activity of IVM in vitro against *F. hepatica* eggs when used in combination with diaryl dichalcogenides [25]. In addition, IVM has been identified as a potential regulator of P-glycoprotein (P-gp), suggesting its important role in combating multidrug resistance. As a result, some researchers are suggesting the proactive use of IVM to help mitigate the development of resistance over time [21,31,55].

Eosinophilia is a hallmark of parasitic infections, including fascioliasis, and reflects the host’s immune response to the parasite [4]. This elevation can benefit the host during Fasciola infection by reducing IL-10 production by specific CD4+ T cells and enhancing eosinophil degranulation triggered by specific antibodies [56]. Prior research indicated that the eosinophil count serves not only as a marker for parasitic infections and underscores the immune system’s effectiveness in fighting the illness but also serves as a predictor of a positive treatment outcome [34].

In this study, patients with high eosinophil counts at the beginning of treatment were more likely to achieve complete recovery. This was demonstrated through regression analyses that examined the relationship between treatment response indicators and complete responses. The significant association between high baseline eosinophil counts and complete recovery (*p* < 0.001) was a key finding of the study. The observed correlation implies that individuals with stronger immune responses may experience improved treatment results, likely because of better parasite elimination. This further reinforces the therapeutic benefits of combining TCBZ and IVM. This finding is especially important and is consistent with earlier studies [7,57].

This study demonstrated promising results for treating fascioliasis by combining TCBZ and IVM, achieving high cure rates. However, several limitations were identified. The small sample size is one of these limitations; however, the disease has a seasonal pattern in the studied area, with difficulty in recruiting large numbers over a longer duration. Additionally, there is a lack of parasitological response, like egg counts, likely due to the disease’s acute nature in most cases. The study was conducted at a single center, which limits the generalizability of the findings. There was also a lack of pharmacokinetic data for the TCBZ and IVM combination to study adequately if there is a synergistic effect. In addition, the study did not include a treatment group for IVM monotherapy. However, the use of IVN monotherapy may raise ethical concerns, as no randomized trials have currently proved the efficacy of IVM in treating human fascioliasis. Therefore, the non-randomized design and small sample size highlight the need for larger, randomized trials to confirm these findings and explore the benefits of combination therapy or IVM monotherapy in diverse patient populations. Future research should focus on longer follow-up periods, multicenter studies to assess resistance patterns, evaluation of the viability of ova shed following combination therapy, and cost-effectiveness evaluations.

## 5. Conclusions

TCBZ is the primary treatment for fascioliasis according to the WHO, but growing resistance and low cure rates prompt the need for alternative therapies. This study provides a promising result of the efficacy of combined TCBZ and IVM for treating fascioliasis in humans, suggesting a possible boosting effect in areas of high TCBZ failure. Combination therapy showed significantly better results, with a complete response rate of 53.3%, compared with 26.2% for TCBZ monotherapy. These effects were evident in clinical, laboratory, and radiological parameters, including improvements in eosinophil counts and hepatic lesions. Further larger randomized trials will be required to confirm these findings and explore the benefits of combination therapy in diverse patient populations.

## Figures and Tables

**Table 1:** Baseline characteristics of the included patients.

Items	Total *n* = 136 (*n*, %)	TCBZ (*n* = 65) (*n*, %)	TCBZ + IVM (*n* = 71) (*n*, %)	*p*-Value
**Gender:**				
Females	77 (56.6)	33 (44)	44 (62)	0.188
Males	59 (43.4)	32 (27)	27 (38)	
**Age (years) (mean ± SD)**	33.6 ± 17.3	31.6 ± 18.2	35.5 ± 16.4	0.195
**Residence:**				
Rural	100 (73.5)	46 (54)	54 (76.1)	0.485
Urban	36 (26.5)	19 (17)	17 (23.9)	
**Chronic fascioliasis**	17 (12.5)	9 (13.8)	8 (11.3)	0.650
***Fasciola* antibody levels (mean)**	890.6 ± 390.3	960 ± 396	827 ± 376.7	0.047 *

* Significant *p*-value.

**Table 2:** Comparison of clinical, radiological, and laboratory data among the treatment groups before and after treatment.

**Items**	**Before** **TCBZ (*n* = 65)** **(*n*, %)**	**After TCBZ** **(*n* = 65)** **(*n*, %)**	***p*** **-Value**	**Before** **TCBZ + IVM** **(*n* = 71)** **(*n*, %)**	**After TCBZ + IVM** **(*n* = 71)** **(*n*, %)**	***p*** **-Value**
**Fever**	44 (67.7)	5 (7.7)	0.000 *	32 (45.1)	0	<0.001 *
**Abdominal pain**	63 (96.9)	8 (12.3)	0.000 *	71 (100)	3 (4.2)	<0.001 *
**Ascites**	7 (10.8)	0	0.016 *	4 (5.6)	0	0.125
**HFL**	25 (38.5)	8 (12.3)	0.000 *	41 (57.7)	6 (8.5)	<0.001 *
**Presence of ova in stool**	6 (9.2)	1 (1.5)	0.063	4 (5.6)	1 (1.4)	0.250
**ALT (IU/L) (median)**	35 (28)	30 (12)	0.000 *	31 (20)	25 (12)	<0.001 *
**AST (IU/L) (median)**	32 (28.5)	26 (12)	0.000 *	28 (19)	22 (11)	<0.001 *
**WBCs cells/μL (median)**	10,500 (8830)	8600 (3355)	0.000 *	10,500 (6140)	7430 (3020)	<0.001 *
**Eosinophilic count** **(median) cells/μL**	3576 (5717)	970 (1854.5)	0.000 *	3250 (6500)	400 (400)	<0.001 *
**Eosinophilic percent** **(median)**	36 (29)	13 (17.8)	0.000 *	30 (37.5)	6 (6)	<0.001 *

HFL: hepatic focal lesion, ALT: alanine aminotransferase, AST: aspartate aminotransferase, WBCs: white blood cells. * Significant *p*-value.

**Table 3:** Comparative analysis of response patterns between treatment groups.

**Items**	**TCBZ** **(*n* = 65)** **(*n*, %)**	**TCBZ + IVM** **(*n* = 71)** **(*n*, %)**	***p-*** **Value**
**Clinical response**	56 (86.2)	68 (95.8)	0.048 *
**Eosinophilic response**	21 (32.3)	50 (70.4)	<0.001 *
**Radiological response**	18 (27.7)	35 (49.3)	0.036 *
**Complete response**	17 (26.2)	40 (53.3)	<0.001 *

* Significant *p*-value.

**Table 4:** Factors associated with complete response.

**Variables**	***p*** **-Value**	**OR**	**CI** **Upper Level**	**CI** **Lower Level**
**Age**	0.234	0.985	0.961	1.010
**Gender**	0.841	0.915	0.382	2.189
**Residence**	0.899	0.938	0.351	2.509
**Duration of symptoms**	0.297	0.992	0.976	1.007
**HFL at baseline**	0.330	0.648	0.271	1.552
**High eosinophil counts at baseline**	0.009 *	1.000	1.000	1.000
**Treatment type**	0.168	1.860	0.770	4.494

HFL: hepatic focal lesion, * Significant *p*-value < 0.05.

## Data Availability

The data presented in this study are available from the corresponding author on reasonable request due to the ethical concern of patients’ privacy.

## Abbreviations

TCBZ	Triclabendazole
IVM	Ivermectin
NTDs	Neglected tropical diseases
WHO	World Health Organization
HFL	Hepatic focal lesion
